# Redução no Número de Pacientes com Síndrome Coronariana Aguda Suspeita e Confirmada nos Primeiros Meses da Pandemia da Covid-19: Análise de uma Rede Brasileira

**DOI:** 10.36660/abc.20200873

**Published:** 2021-05-06

**Authors:** Pedro Gabriel Melo de Barros e Silva, Ana Amaral Ferreira Dutra, Adriana Bertolami Manfredi, Pedro Paulo Nogueres Sampaio, Celso Musa Correa, Hemilo Borba Griz, Daniel Setta, Valter Furlan

**Affiliations:** 1 Hospital Samaritano Paulista São PauloSP Brasil Hospital Samaritano Paulista, São Paulo, SP Brasil; 2 Cardiologia Americas São PauloSP Brasil Cardiologia Americas, São Paulo, SP Brasil; 3 Hospital Pró-Cardíaco Rio de JaneiroRJ Brasil Hospital Pró-Cardíaco, Rio de Janeiro, RJ Brasil; 4 Hospital Alvorada Moema São PauloSP Brasil Hospital Alvorada Moema, São Paulo, SP Brasil; 5 Hospital Samaritano Botafogo Rio de JaneiroRJ Brasil Hospital Samaritano Botafogo, Rio de Janeiro, RJ - Brasil; 6 Americas Medical City Rio de JaneiroRJ Brasil Americas Medical City, Rio de Janeiro, RJ Brasil; 7 Hospital Santa Joana Recife RecifePE Brasil Hospital Santa Joana Recife, Recife, PE - Brasil

**Keywords:** Hospitais Públicos, Dor no Peito, Hospitais Privados, Síndrome Coronariana Aguda, Pandemia, Epidemiologia, Estudo Comparativo

## Introdução

Os primeiros relatos de infecções pela síndrome respiratória aguda grave coronavírus 2 (SARS-CoV-2) ocorreram em dezembro de 2019 em Wuhan, China.^1^^,^^2^ A doença (denominada doença coronavírus-2019, Covid-19) se espalhou rapidamente pelo mundo e, em 11 de março de 2020, a Organização Mundial da Saúde (OMS) declarou estado de pandemia.^1^^,^^2^ O “Lockdown” era uma recomendação comum em países afetados.^3^

Apesar de toda a atenção dada à Covid-19 pelas autoridades de saúde, outras doenças também podem ser impactadas por esta nova circunstância. As taxas de doenças cardiovasculares agudas mudaram em países como Itália e Estados Unidos, com redução nas internações.^4^^–^^6^ Dados nacionais anteriores sobre síndromes coronarianas agudas (SCA) já estão bem e amplamente descritos, mas esses estudos não incluíram o período da Covid-19.^7^^,^^8^ Assim, apesar de o Brasil ter sido o segundo país mais afetado em número de casos de Covid-19,^9^ o impacto nas internações por suspeita ou confirmação de SCA ainda não está bem definido nos sistemas de saúde público e privado brasileiros.

O objetivo deste relatório foi comparar o número de pacientes com suspeita e confirmação de SCA antes e durante os primeiros meses da pandemia de Covid-19 em uma rede de hospitais privados no Brasil.

## Métodos

### Desenho do estudo

Análise de cadastro de pacientes incluídos em um mesmo Protocolo de Dor Torácica em uma rede de 16 hospitais de seis diferentes Estados do Brasil. O estudo foi aprovado pelo conselho de revisão institucional (20710119.4.0000.5533).

### Participantes e variáveis

Em 2019, uma rede privada de hospitais implementou um Protocolo de Dor Torácica com o objetivo de padronizar a investigação e o tratamento de pacientes com suspeita de sintomas de SCA e fornecer métricas para iniciativas de melhoria da qualidade. Os indivíduos foram incluídos no Protocolo de Dor Torácica com base nos seguintes critérios: dor torácica aguda independentemente dos fatores de risco e/ou sintoma equivalente a angina, como falta de ar (dispneia) em pacientes com alto risco cardíaco (idade >65 anos ou histórico de fatores de risco). Os pacientes com diagnóstico confirmado de SCA foram classificados de acordo com a presença ou ausência de supradesnivelamento de ST. Os mesmos critérios foram usados antes e durante a pandemia de Covid-19 para inclusão no protocolo de dor torácica. As opções de tratamento também foram as mesmas nos dois períodos, ou seja, preferência pela intervenção coronária percutânea primária nos casos de infarto com supradesnivelamento do segmento ST (IAMCSST). As únicas diferenças foram relativas ao uso rotineiro de equipamentos de proteção individual (EPI) e o local de investigação, de acordo com a probabilidade de Covid-19, já que os pacientes com sintomas infecciosos ou respiratórios foram avaliados em unidades específicas.^10^ Os desfechos clínicos de mortalidade intra-hospitalar e fração de ejeção reduzida (FE<40%) também foram coletados rotineiramente de todos os pacientes com SCA em ambos os períodos.

As variáveis relacionadas aos primeiros três meses da pandemia de Covid-19 no Brasil (março a maio de 2020) foram comparadas às do mesmo período de 2019, e também aos dois meses imediatamente anteriores ao surto (janeiro e fevereiro de 2020) e à média dos resultados dos últimos 12 meses. Essas diferentes comparações foram escolhidas para que se pudesse avaliar um maior número de casos em um período de observação mais longo e para evitar variações sazonais que podem ocorrer ao longo de diferentes períodos do ano calendário.

### Análises estatísticas

As variáveis categóricas foram apresentadas em frequências absolutas e relativas, enquanto as variáveis contínuas foram descritas como média e desvio-padrão (DP). Os grupos foram comparados pelo teste t para variáveis contínuas e o teste de Qui-quadrado para variáveis categóricas. Os valores de p bicaudais abaixo de 0,05 foram considerados estatisticamente significativos. A análise foi realizada no software R, versão 3.6.1 (R Foundation for Statistical Computing).

## Resultados

### Análise de pacientes com suspeita de SCA antes e durante os primeiros meses da pandemia de Covid-19

**Figura 1:**
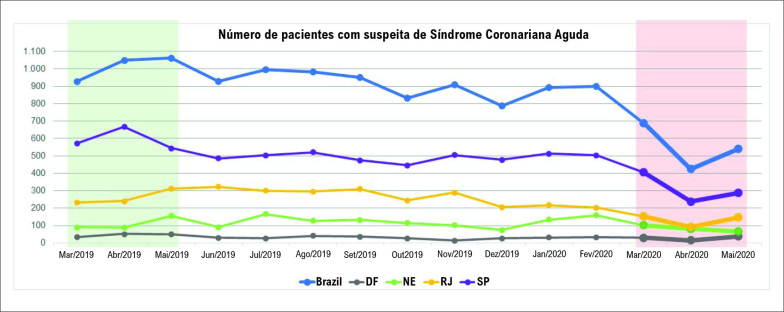
Número de pacientes com suspeita de Síndrome Coronariana Aguda antes e durante os três primeiros meses da pandemia de Covid-19 no Brasil (geral e em quatro regiões diferentes). DF: Distrito Federal (1 hospital); NE: Nordeste (3 hospitais de 3 Estados diferentes: Pernambuco, Rio Grande do Norte e Ceará); RJ: Rio de Janeiro (4 hospitais); SP: São Paulo (8 hospitais).

A idade média (52,9 ± 7,2 vs. 53,2 ± 6,9; p = 0,16) e a porcentagem de mulheres nas amostras (45,3%, 749/1.653 vs. 46,9%, 1.427/3.040; p = 0,29) não mudou comparando-se pacientes com suspeita de SCA nos primeiros meses da Covid-19 com o mesmo período do ano anterior (março a maio de 2019). O número de pacientes atendidos no pronto-socorro com sintomas suspeitos de SCA (e incluídos no Protocolo de Dor Torácica) diminuiu nos primeiros três meses da pandemia (Figura 1). Essa queda foi mais pronunciada nos dois primeiros meses em São Paulo e no Rio de Janeiro, porém mais gradual nos hospitais do Nordeste do Brasil (Figura 1). No Distrito Federal, a curva não apresentou alteração relevante no início da pandemia, mas a análise se limitou a apenas um hospital (Figura 1). No geral, a média mensal de pacientes com suspeita de sintomas de SCA nos primeiros três meses da pandemia reduziu 42,1% em comparação aos 12 meses anteriores (934,0 ± 81,2 vs. 541,3 ± 134,7; p<0,01), 46,6% em comparação aos mesmos três meses em 2019 (1013,3 ± 74,2 vs. 541,3 ± 134,7; p<0,01), e 39,6% em relação a janeiro e fevereiro de 2020 (895,0 ± 4,2 vs. 541,3 ± 134,7; p = 0,03).

### Análise de pacientes com SCA confirmada antes e durante os primeiros meses da pandemia de Covid-19

Comparando os primeiros três meses da pandemia de Covid-19 com a média mensal dos 12 meses anteriores, observou-se redução de 36,5% no número de pacientes com SCA, sendo mais pronunciada em casos de SCA sem supradesnivelamento do segmento ST (Tabela 1). Esses resultados foram semelhantes aos de três relatórios internacionais (Tabela 1). As taxas de mortalidade intra-hospitalar desta rede brasileira nos 12 meses anteriores também foram comparadas às atuais e, ao contrário dos dados do Registro italiano, não foram superiores (Tabela 1). Apesar de não haver aumento na mortalidade, a porcentagem de pacientes que receberam alta com fração de ejeção reduzida (<40%) após SCA foi maior nos primeiros três meses da pandemia quando comparada aos 12 meses anteriores (7,1%, 127/1.777 vs. 11,1%, 34/306; p = 0,02). Durante 15 meses de análise (março de 2019 a maio de 2020), todos os pacientes com SCA receberam terapia antiplaquetária dupla e todos os pacientes com IAMCSST receberam terapia de reperfusão. O tempo médio porta-balão dos 12 meses anteriores não mudou quando comparado aos primeiros meses da pandemia de Covid-19 (70,3 ± 18,1 vs.72,1 ± 19,8; p = 0,60).

**Tabela 1:** Mudanças relativas nos diagnósticos de Síndrome Coronariana Aguda e mortalidade hospitalar antes e durante o período inicial da pandemia de Covid-19 na análise atual e na literatura internacional (Norte da Itália^4^, Kaiser Permanente^5^, Registro Italiano^6^)

	Total SCA^a^	IAMCSST	IAMSSST	Mortalidade no hospital
Norte da Itália^b^	redução de 28%	redução de 24%	redução de 43%	Não disponível
Kaiser Permanente^c^	redução de 48%	redução de 40%	redução de 49%	Não disponível
Registro italiano^d^	redução de 48.4%	redução de 26.5%	redução de 65.1%	RR_geral_= 3.6 (2.0–6.4) RR_IAMCSST_= 3.3 (1.7–6.6)
Rede brasileira^e^	redução de 36.5%^e^	redução de 28.9%^e^	redução de 39.5%^e^	RR_geral_= 0.85 (0.4-1.7)^e^ RR_IAMCSST_= 1.2 (0.3-4.0)^e^

SCA: Síndrome Coronariana Aguda; IAMCSST: infarto do miocárdio com supradesnivelamento do segmento ST; IAMSSST: infarto do miocárdio sem supradesnivelamento do segmento ST; RR: Razão de risco.

a Nos relatórios da Kaiser Permanente e do Registro Italiano, apenas o infarto agudo do miocárdio foi avaliado.

b Análise retrospectiva de pacientes consecutivos admitidos por síndrome coronariana aguda em 15 hospitais no norte da Itália, comparando fevereiro e março a dois períodos de controle: período correspondente no ano anterior (2019) e um período anterior no mesmo ano (2020)^4^. Nós relatamos a média de ambas as análises.

c A comparação foi baseada em um banco de dados de um sistema de prestação de cuidados de saúde de janeiro a março de 2020, em comparação com os dados de abril de 2020^5^.

d Dados baseados em uma pesquisa nacional sobre admissões por infarto agudo do miocárdio em unidades de cuidados coronários na Itália em um período de uma semana durante o surto de Covid-19, e em comparação com a semana equivalente em 2019^6^.

e Comparação entre a média dos primeiros três meses da pandemia de Covid-19 (94 SCA por mês; 21 IAMCSST por mês; 53,3 IAMSSST por mês) e a média dos 12 meses anteriores (148,1 SCA por mês; 29,5 IAMCSST por mês; 88,1 IAMSSST por mês). No geral, a taxa de mortalidade foi de 3,4% (61/1,777) nos 12 meses anteriores e 2,9% nos primeiros três meses da pandemia (9/306). A mortalidade foi de 3,9% entre os pacientes com IAMCSST (14/354) nos 12 meses anteriores e 4,7% (3/63) nos primeiros três meses da pandemia.

## Discussão

Este artigo teve como objetivo avaliar os números relacionados à SCA no Brasil, incluindo o número de pacientes que procuram atendimento médico e a taxa de diagnósticos confirmados e principais desfechos clínicos. Encontramos redução não apenas no número de pacientes com diagnóstico confirmado de SCA, mas também no número de pacientes que procuram atendimento médico por suspeita de SCA. A maioria das publicações anteriores focava pacientes com diagnóstico confirmado, e não suspeita clínica.^4^^–^^6^ Isso variou de acordo com a região, sendo mais pronunciado nos dois primeiros meses em São Paulo e no Rio de Janeiro, com declínio gradual no Nordeste do Brasil. Provavelmente, isso decorre do número total de casos de Covid-19, que foi mais pronunciado em São Paulo, especialmente na fase inicial da pandemia.

Os dados identificados nesta rede de 16 hospitais de seis Estados diferentes reforçam os achados de relatórios internacionais anteriores sobre redução de diagnósticos de SCA intra-hospitalar. A queda no número de casos confirmados pode indicar que os casos mais graves que não procuraram imediatamente atendimento médico podem ter apresentado resultado fatal fora do hospital. Isso foi identificado em publicações anteriores,^11^^,^^12^ mas não pôde ser avaliado em nosso banco de dados nacional, que inclui apenas informações intra-hospitalares. Além disso, a redução >40% no número de pacientes que procuraram atendimento médico nos primeiros meses da pandemia foi associada a uma taxa maior de pacientes com fração de ejeção baixa apesar de cuidados médicos adequados nas métricas de qualidade, indicando que a população afetada por SCA na fase pandêmica era composta por pacientes mais graves. Isso pode ser um indício de que os pacientes com apresentações menos graves e transitórias podem não ter procurado atendimento médico na fase inicial da pandemia por medo de contaminação. Assim, a queda drástica nos casos de SCA pode ser justificada não apenas por casos mais críticos com desfechos potencialmente fatais fora dos hospitais, mas também por pacientes com manifestações menos graves que normalmente buscariam avaliação médica, mas não o fizeram no contexto da pandemia. Esta última situação representa o grupo de pacientes que podem ter sobrevivido ao evento agudo, mas estariam em maior risco no futuro devido à falta de tratamento.

Em suma, o número pequeno de pacientes admitidos para avaliação de emergência traz preocupação em relação aos pacientes que tiveram um evento de SCA em casa e poderiam apresentar piores desfechos em curto e longo prazo. Nossos achados, juntamente com dados anteriores da literatura internacional, reforçam a necessidade de procurar atendimento médico em casos de suspeita de eventos cardiovasculares, mesmo durante um período de lockdown, como no início da pandemia de Covid-19.

### Limitações

Este é um relatório baseado em um banco de dados específico desenvolvido para monitorar iniciativas de melhoria relacionadas ao Protocolo de Dor Torácica. Informações como duração dos sintomas e características basais dos pacientes, exceto idade e sexo, não foram incluídas nos registros e não puderam ser avaliadas. Finalmente, a falta de informações sobre infecção concomitante ou recente por Covid-19 não permite uma análise de uma possível relação com menor fração de ejeção após infarto do miocárdio em pacientes com histórico recente de infecção por SARS-CoV-2.

## Conclusão

Em uma rede de hospitais no Brasil, identificamos uma redução de mais de 40% em pacientes com suspeita de SCA e 36,5% nas internações por SCA confirmada quando comparamos os primeiros meses da pandemia de Covid-19 com a média dos meses anteriores. Esses achados alertam para um número menor de pacientes que procuram o pronto-socorro durante da pandemia de Covid-19 no Brasil. As sociedades médicas nacionais e os sistemas de saúde devem monitorar as potenciais consequências adversas na saúde pública, como um aumento nos casos de insuficiência cardíaca após infarto do miocárdio.
