# Supplementary material for: Redução no Número de Pacientes com Síndrome Coronariana Aguda Suspeita e Confirmada nos Primeiros Meses da Pandemia da Covid-19: Análise de uma Rede Brasileira
Source: Arq Bras Cardiol. 2021 May 6;116(5):1003–6. [Article in Portuguese] doi: 10.36660/abc.20200873 (PMC8121460; doi:10.36660/abc.20200873)
Supplement: Translation

# Translation: Reduction in the Number of Patients with Suspected and Confirmed Acute Coronary Syndrome during the early months of the Covid-19 Pandemic: Analysis of a Brazilian Network

**Authors:** Pedro Gabriel Melo de Barros e Silva, Ana Amaral Ferreira Dutra, Adriana Bertolami Manfredi, Pedro Paulo Nogueres Sampaio, Celso Musa Correa, Hemilo Borba Griz, Daniel Setta, Valter Furlan

**Affiliations:** 1 São PauloSP Brazil Hospital Samaritan Paulista, São Paulo, SP - Brazil; 2 São PauloSP Brazil Cardiologia Americas, São Paulo, SP - Brazil; 3 Rio de JaneiroRJ Brazil Hospital Pró-Cardíaco, Rio de Janeiro, RJ - Brazil; 4 São PauloSP Brazil Hospital Alvorada Moema, São Paulo, SP - Brazil; 5 Rio de JaneiroRJ Brazil Hospital Samaritano Botafogo, Rio de Janeiro, RJ - Brazil; 6 Rio de JaneiroRJ Brazil Americas Medical City, Rio de Janeiro, RJ - Brazil; 7 RecifePE Brazil Hospital Santa Joana Recife, Recife, PE - Brazil

**Keywords:** Hospitals, Public, Chest Pain, Hospitals, Private, Acute Coronary Syndrome Pandemics, Epidemiology, Comparative Study

## Introduction

The first reports of infections by the severe acute respiratory syndrome coronavirus 2 (SARS-CoV-2) occurred in December of 2019 in Wuhan, China.^1^^,^^2^ This disease (named as coronavirus disease-2019, Covid-19) rapidly spread globally and, on March 11, 2020, the World Health Organization (WHO) declared the state of pandemic.^1^^,^^2^ Lockdown was a common recommendation for countries affected by the outbreak.^3^

Despite all the attention put on Covid-19 by the health authorities, other diseases could also be impacted by this new circumstance. Rates of acute cardiovascular diseases changed in countries such as Italy and United States, with a reduction in hospital admissions.^4^^–^^6^ Previous national data regarding acute coronary syndrome (ACS) is already well and largely described, but these studies did not include the Covid-19 period.^7^^,^^8^ Thus, despite the fact that Brazil was the second most affected country when it comes to number of Covid-19 cases,^9^ the impact in hospital admissions due to suspected or confirmed ACS is still not well defined in both private and public Brazilian healthcare systems.

The objective of this report was to compare the number of patients with suspected and confirmed ACS before and during the early months of the Covid-19 pandemic in a network of private hospitals in Brazil.

## Methods

### Study design

Analysis of a registry of patients included in the same Chest Pain Protocol in a network of 16 hospitals in 6 different States in Brazil. The study was approved by the institutional review board (20710119.4.0000.5533).

### Study participants and study variables

In 2019, a private network of hospitals implemented a Chest Pain Protocol aiming to standardize investigation and treatment of patients with suspected symptoms of ACS and to provide metrics for quality improvement initiatives. Subjects were included in the Chest Pain Protocol based on the following criteria: acute chest pain regardless of risk factors and/or anginal equivalent symptom, such as shortness of breath (dyspnea), in patients at high cardiac risk (age >65 years old or history of risk factors). Patients with a confirmed diagnosis of ACS were classified according to presence or absence of ST-elevation. The same criteria were used before and during the Covid-19 outbreak for inclusion in the Chest Pain Protocol. The treatment options were also the same in both periods, that is, preference for primary percutaneous coronary intervention in cases of ST-elevation myocardial infarction (STEMI). The only differences were regarding the routine use of personal protective equipment (PPE) and the location for investigation according to Covid-19 probability, since patients with infectious or respiratory symptoms were evaluated in specific units.^10^ Clinical outcomes of in-hospital mortality and low ejection fraction (EF<40%) were also routinely collected from all ACS patients in both periods.

The variables related to the first three months of Covid-19 outbreak in Brazil (March to May, 2020) were compared to those of the same period in 2019, and also to the two months just before the Covid-19 outbreak in Brazil (January and February, 2020) and the average of previous 12-month results. These different comparisons were chosen so one could assess a larger number of cases in a longer period of observation and also to avoid seasonal variations that may occur along different periods of a calendar year.

### Statistical analyses

Categorical variables were reported as absolute and relative frequencies, while continuous variables were described as mean and standard deviation (SD). Groups were compared by the t-test for continuous variables and the Chi-square test for categorical variables. P values were two-tailed, and values below 0.05 were considered statistically significant. The analysis was performed using the R software, version 3.6.1 (R Foundation for Statistical Computing).

## Results

### Analysis of patients with Suspected ACS before and during the early months of the Covid-19 pandemic

The mean age (52.9 ± 7.2 vs. 53.2 ± 6.9; P =0.16) and percentage of women in the samples (45.3%, 749/1,653 vs. 46.9%, 1,427/3,040; P= 0.29) did not change comparing patients with suspected ACS in the early months of Covid-19 with the same period in the previous year (March to May, 2019). The number of patients seen to the emergency department with suspected ACS symptoms (and included in the Chest Pain Protocol) dropped in the first three months of the pandemic (Figure 1). This decrease was more pronounced in the first two months in Sao Paulo and Rio de Janeiro, while it was more gradual in the hospitals from the Northeast of Brazil (Figure 1). In the Federal District, the curve did not show a relevant change during the start of the pandemic, but the analysis was limited to only one hospital (Figure 1). Overall, the monthly average of patients with suspected symptoms of ACS in the first three months of the pandemic reduced 42.1% compared to the previous 12 months (934.0 ± 81.2 vs. 541.3 ± 134.7; p < 0.01), 46.6% compared to the same three months in 2019 (1013.3 ± 74.2 vs. 541.3 ± 134.7; p < 0.01), and 39.6% compared to January and February of 2020 (895.0 ± 4.2 vs. 541.3 ± 134.7; p = 0.03).

**Figure 1:** Number of patients with suspected Acute Coronary Syndrome before and during the first three months of the Covid-19 outbreak in Brazil (overall and in four different regions). DF: Federal District (1 hospital); NE: Northeast (3 hospitals from 3 different States: Pernambuco, Rio Grande do Norte and Ceará); RJ: Rio de Janeiro (4 hospitals); SP: São Paulo (8 hospitals).

### Analysis of Patients with Confirmed ACS before and during the Early Months of the Covid-19 pandemic

Comparing the first three months of the Covid-19 outbreak with the monthly average in the 12 previous months, a reduction of 36.5% in the number of ACS patients was seen, being more pronounced in non-ST elevation ACS (Table 1). These results were similar to those of three international reports (Table 1). The rates of In-hospital mortality in this Brazilian network in the 12 previous months were also checked against the current ones and, unlike the Italian Registry's data, they were not higher (Table 1). In spite of no increase in mortality, the percentage of patients being discharged after an ACS with low ejection fraction was higher in the first three months of the pandemics when compared to the 12 previous months (7.1%, 127/1,777 vs. 11.1%, 34/306; p = 0.02). During 15 months of analysis (March, 2019 to May, 2020), all eligible patients with ACS received double antiplatelet therapy and all STEMI patients received reperfusion therapy. The mean door-to-balloon time of the 12 previous months did not change when checked against that of the early months of the Covid-19 pandemic (70.3±18.1 vs.72.1±19.8; p=0.60).

**Table 1:** Relative changes in Acute Coronary Syndrome diagnoses and in-hospital mortality before and during initial period of Covid-19 outbreak in the current analysis and in the international literature (North Italy^4^, Kaiser Permanente^5^, Italian Registry^6^)

	Total ACS^a^	STEMI	NSTEMI	In-hospital mortality
North Italy^b^	28% reduction	24% reduction	43% reduction	Not available
Kaiser Permanente^c^	48% reduction	40% reduction	49% reduction	Not available
Italian Registry^d^	48.4% reduction	26.5% reduction	65.1% reduction	RR_overall_= 3.6 (2.0–6.4) RR_STEMI_= 3.3 (1.7–6.6)
Brazilian Network^e^	36.5% reduction^e^	28.9% reduction^e^	39.5% reduction^e^	RR_overall_= 0.85 (0.4-1.7)^e^ RR_STEMI_= 1.2 (0.3-4.0)^e^

ACS: Acute Coronary Syndrome; STEMI: ST-Elevation Myocardial Infarction; NSTEMI: Non-ST-Elevation Myocardial infarction; RR: Risk Ratio.

a In the reports from Kaiser Permanente and Italian Registry, only acute myocardial infarction was evaluated.

b Retrospective analysis of consecutive patients who were admitted for acute coronary syndrome at 15 hospitals in northern Italy, comparing February and March to two control periods: a corresponding period in the previous year (2019) and an earlier period in the same year (2020)^4^. We reported the average from both analyses.

c The comparison reported was based on a database of a health care delivery system from January through March 2020, compared to data from April 2020^5^.

d Data based on a nationwide survey regarding admissions for acute myocardial infarction at Italian coronary care units in a one-week period during the Covid-19 outbreak, and compared with the equivalent week in 2019^6^.

e Comparison between the average of the first three months of the Covid-19 pandemic (94 ACS per month; 21 STEMI per month; 53.3 NSTEMI per month) and the average of the previous 12 months (148.1 ACS per month; 29.5 STEMI per month; 88.1 NSTEMI per month). Overall, the mortality rate was 3.4% (61/1777) in the previous 12 months and 2.9% in the first three months of the pandemic (9/306). The mortality was 3.9% among STEMI patients (14/354) in the previous 12 months and 4.7% (3/63) in the first three months of the pandemic.

## Discussion

This paper aimed to evaluate the numbers related to ACS in Brazil, including the number of patients seeking medical care and the rate of confirmed diagnosis and major clinical outcomes. We found a reduction not only in the number of patients with confirmed ACS diagnosis, but also in the number of patients seeking medical care due to suspected ACS. Most of the previous publications were focused on patients with confirmed diagnosis, not on clinical suspicion.^4^^–^^6^ This varied according to the region, being more pronounced in the first two months in Sao Paulo and Rio de Janeiro, with a more gradual decline in the northeast of Brazil. This probably stems from the total number of cases of Covid-19, which was more pronounced in Sao Paulo, especially in the early phase of the pandemic.

The data identified in this network of 16 hospitals from six different States reinforce the findings from previous international reports about reduction of in-hospital ACS diagnosis. The drop in the number of confirmed cases may indicate that more severe cases that did not immediately seek medical care may have been fatal outcomes out of the hospital. This was identified in previous publications,^11^^,^^12^ but could not be assessed in our national database, since it includes only in-hospital information. In addition, the >40% reduction in the number of patients who sought medical care in the early months of the pandemic was associated to a higher rate of patients with low ejection fraction despite adequate medical care, indicating that the population affected by ACS in the pandemic phase were composed of more severe patients. This may indicate that patients with less severe and transitory presentations may not have sought medical care in the early phase of the pandemic for fear of contamination. Thus, the drastic drop in ACS cases may be justified not only by more critical cases with potentially fatal outcomes out of the hospitals, but also by patients with less severe manifestations that would normally seek medical evaluation, but did not do so in the context of the pandemic. This last situation accounts for the group of patients that may have survived the acute event, but they would be at a greater risk in the future due to the lack of treatment.

In summary, the small number of patients admitted for emergency evaluation raises a concern as to patients who had an ACS event at home and could present worse outcomes in the short and long terms. Our findings, along with previous data from the international literature, reinforce how necessary it is to seek medical care in suspected cardiovascular events even during a lockdown period like the start of the Covid-19 pandemic.

### Limitations

This is a report based on a specific database developed for monitoring quality improvement initiatives related to the Chest Pain Protocol. Information such as symptom duration and baseline characteristics of patients, other than age and gender, were not included in the registries and could not be assessed. Finally, the lack of information about concomitant or recent Covid-19 infection does not allow an analysis of a possible relation with lower ejection fraction after myocardial infarction in patients with recent history of SARS-CoV-2 infection.

## Conclusion

In a network of hospitals in Brazil, we identified a reduction of more than 40% of patients with suspected ACS and 36.5% in admissions due to confirmed ACS when comparing the early months of the Covid-19 pandemic to the average of previous months. These findings raise an alert of a smaller number of patients seeking emergency departments during the Covid-19 outbreak in Brazil. National medical societies and healthcare systems should monitor potential adverse consequences in the public health such as an increase in cases of heart failure following myocardial infarction.
